# Polyphenol-Rich Cinnamon Bud Extract Affects Ataxin-3 Aggregation and Ameliorates SCA3 Phenotypes Through a Dual Anti-Amyloidogenic and Antioxidant Mechanism

**DOI:** 10.3390/molecules31142510

**Published:** 2026-07-17

**Authors:** Barbara Sciandrone, Roberta Pensotti, Diletta Ami, Alessia Saponara, Riccardo Campanile, Valeria Cassina, Antonino Natalello, Alessandro Palmioli, Cristina Airoldi, Maria Elena Regonesi

**Affiliations:** 1 Department of Biotechnology and Biosciences BtBs, University of Milano-Bicocca, Piazza della Scienza 2, 20126 Milan, Italy; barbara.sciandrone@unimib.it (B.S.); r.pensotti1@campus.unimib.it (R.P.); diletta.ami@unimib.it (D.A.); antonino.natalello@unimib.it (A.N.); alessandro.palmioli@unimib.it (A.P.); cristina.airoldi@unimib.it (C.A.); 2 School of Medicine and Surgery, University of Milano-Bicocca, Via Raoul Follereau 3, 20854 Vedano al Lambro, Italy; a.saponara2@campus.unimib.it (A.S.); r.campanile@campus.unimib.it (R.C.); valeria.cassina@unimib.it (V.C.); 3 BioNanoMedicine Center NANOMIB, University of Milano-Bicocca, 20854 Vedano al Lambro, Italy; 4 NeuroMI, Milan Center for Neuroscience, University of Milano-Bicocca, 20126 Milan, Italy

**Keywords:** antioxidant, *Caenorhabditis elegans*, cinnamon buds, neurodegeneration, nutrition

## Abstract

Aberrant self-assembly of ataxin-3 (ATX3) into amyloid aggregates is a key pathological event in spinocerebellar ataxia type 3 (SCA3). Bioactive nutraceutical compounds, particularly polyphenols, have emerged as promising candidates for targeting protein aggregation and cellular stress responses associated with neurodegenerative disorders. Here, we investigated cinnamon bud extract as a natural source of neuroprotective molecules, focusing on its total extract (Etot) and two bioactive fractions: a polyphenol-enriched fraction (Fr. B) and a cinnamaldehyde-rich fraction (Fr. C). By integrating biochemical and biophysical techniques, we demonstrate that cinnamon-derived compounds modulate ATX3 aggregation by reducing the formation of β-sheet-rich amyloid assemblies and promoting the generation of SDS-resistant, soluble, structurally distinct, non-fibrillar species. NMR profiling identified flavonoids, cinnamaldehyde, and cinnamic acid as key ATX3-interacting molecules, supporting their contribution to the anti-amyloidogenic activity of the extract. Moreover, in a *Caenorhabditis elegans* SCA3 model, Etot and Fr. B improved locomotor defects and enhanced resistance to oxidative and thermal stress, indicating broader cytoprotective effects beyond direct aggregation modulation. Overall, these findings highlight cinnamon bud extract, particularly its polyphenol-rich fraction, as a promising nutraceutical source of bioactive compounds with potential neuroprotective properties and provide a basis for further investigation of nutraceutical strategies targeting polyglutamine-related neurodegenerative diseases.

## 1. Introduction

“Let food be thy medicine and medicine be thy food”. With this sentence, Hippocrates highlights the long-recognized importance of nutrition in maintaining human health [1,2]. This concept remains highly relevant in modern scientific research, which is increasingly focused on the identification and characterization of bioactive compounds in food. These natural compounds, often derived from plant-based dietary sources, are gaining considerable attention for their potential to promote health and prevent chronic diseases [3,4,5]. The interest is particularly timely for the challenges posed by an aging global population, where evidence-based interventions are urgently needed to counteract frailty and age-related pathologies [6,7].

Among the most extensively studied bioactive compounds are phytochemicals, particularly polyphenols, which are well known for their wide spectrum of biological activities. These include antimicrobial [8,9], anticancer [10,11], anti-inflammatory [12,13], anti-aging [14,15] and neuroprotective effects [16,17,18]. The beneficial health properties of polyphenols are largely attributed to their potent antioxidant activities, which enables them to neutralize reactive oxygen species (ROS) and reduce oxidative stress, a key factor implicated in the pathogenesis of neurodegenerative disorders [19,20,21]. Their unique chemical structures, characterized by multiple phenolic rings, confer strong radical-scavenging capacities [21].

Polyphenols are abundantly found in a variety of commonly consumed foods, including fruits (particularly wild berries and apples), vegetables, legumes, dark chocolate, black tea, coffee, and spices such as cinnamon, ginger, and cumin [2,5,22,23]. Regular consumption of these polyphenol-rich foods is therefore regarded as a key dietary strategy for maintaining health and preventing disease. Among these, *Cinnamomum cassia* has emerged as a particularly interesting source of bioactive polyphenols. In particular, its bark and buds, widely used in traditional Asian cuisine, are rich in procyanidins and cinnamaldehydes, compounds associated with various health-promoting effects [4,24,25]. While much of the previous research has focused on the pro-longevity and neuroprotective properties of the burk [26,27], recent interest has shifted toward the unopened flowers (buds) of the cinnamon tree. In previous studies, the metabolic profile of these buds was characterized using nuclear magnetic resonance (NMR) spectroscopy [28] and ultra performance liquid chromatography coupled with high-resolution mass spectrometry (UPLC-HR-MS) [29]. Specifically, non-targeted 1H-NMR profiling of the hydroalcoholic extracts highlighted a complex chemical framework. The aromatic fractions are heavily dominated by (E)-cinnamaldehyde and its structural congeners (such as 2-methoxycinnamaldehyde and 2-hydroxycinnamaldehyde), alongside organic acids like benzoic, cinnamic, and shikimic acids, and primary metabolites including sucrose, glucose, fructose, and choline. When compared to the bark extracts via UPLC-HRMS analysis of the polyphenol-enriched fractions, the buds present a highly distinct and diversified polyphenolic fingerprint. While sharing common compounds like catechin and epicatechin, the bud extract is uniquely enriched with a wider cascade of high-molecular-weight oligomeric tannins, specifically extending from B-type procyanidin dimers and trimers up to hexamers and heptamers. Furthermore, the buds stand out for their abundance of glycosylated flavonoids, phenyl glycosides, and organic conjugates. Notable exclusive or characteristic markers identified in the buds include specific flavonol glycosides such as quercetin 3-vicianoside, isoquercitrin, avicularin, astragalin, quercitrin, and juglanin, alongside kaempferol derivatives, the chalcone poncirin chalcone, and other specific glycosides like rosavin, cichorioside L, and phenylethyl primeveroside.

The occurrence of these structurally complex polyphenols is particularly noteworthy because they have been associated with a wide range of biological activities and may retain their bioactivity despite the extensive metabolic transformations that often limit the therapeutic potential of dietary polyphenols [30,31]. Subsequent *in vitro* studies demonstrated that cinnamon bud extract exhibits significant antioxidant and neuroprotective activities, including the ability to inhibit Aβ-peptide and tau aggregation, two critical pathogenic events in Alzheimer’s disease [28,29,32]. Collectively, these findings highlight cinnamon buds as a promising, yet underexplored, natural source of nutraceutical compounds with potential neuroprotective properties and relevance for the prevention of neurodegenerative disorders.

In light of these findings, we further investigated the anti-amyloidogenic capacity of cinnamon bud extract in the context of spinocerebellar ataxia type 3 (SCA3), the most common subtype of autosomal dominant cerebellar ataxias and a member of the polyglutamine (polyQ) disorder family. These neurodegenerative diseases are characterized by the abnormal expansion of CAG trinucleotide repeats within specific coding regions [33,34,35]. In SCA3, the pathogenic CAG expansion occurs in the ATXN3 gene, which encodes the deubiquitinating enzyme ataxin-3 (ATX3) protein. When the polyglutamine tract exceeds approximately 55 glutamine residues, ATX3 undergoes misfolding and adopts β-sheet-rich conformations [36]. These structural changes promote the self-association of the protein into the oligomeric intermediates and insoluble amyloid fibrils that accumulate within neuronal nuclei, particularly in the cerebellum and brainstem [37,38,39]. These aggregates compromise cellular integrity by disrupting critical cellular processes, including transcriptional regulation, proteostasis, and mitochondrial function, ultimately contributing to neuronal dysfunction and cell death [40,41]. In addition to proteotoxicity, oxidative stress has been increasingly recognized as a major contributor to SCA3 pathogenesis [42,43]. The expanded ATX3 protein disrupts mitochondrial homeostasis, leading to elevated ROS production and a chronic oxidative environment that damages lipids, DNA, and proteins [42,44,45]. This redox imbalance also impairs cellular clearance mechanisms, such as the ubiquitin–proteasome system and autophagy, exacerbating the accumulation of misfolded proteins and enhancing neuronal vulnerability [46,47]. Therefore, targeting both protein misfolding and oxidative stress represents a promising therapeutic strategy in SCA3. For this reason, we explored the anti-amyloidogenic properties of cinnamon bud extract and of its polyphenolic-enrich fraction on ATX3 aggregation using a combination of *in vitro* biochemical and biophysical approaches, as well as assessing their potential beneficial effects *in vivo* using the model organism *Caenorhabditis elegans*. To this end, we employed the N2 wild type strain and two transgenic strains expressing either a normal or an expanded human ATX3 variant in the nervous system. This experimental design allowed us to evaluate not only the ability of the extract to affect aggregation and mitigate SCA3-associated phenotypes but also to examine its potential to reduce ATX3-induced toxicity through modulation of oxidative stress.

## 2. Results and Discussion

### 2.1. Anti-Amyloidogenic Effects of Cinnamon Bud Extract and Its Principal Components on Ataxin-3 Aggregation

**Figure 1:**
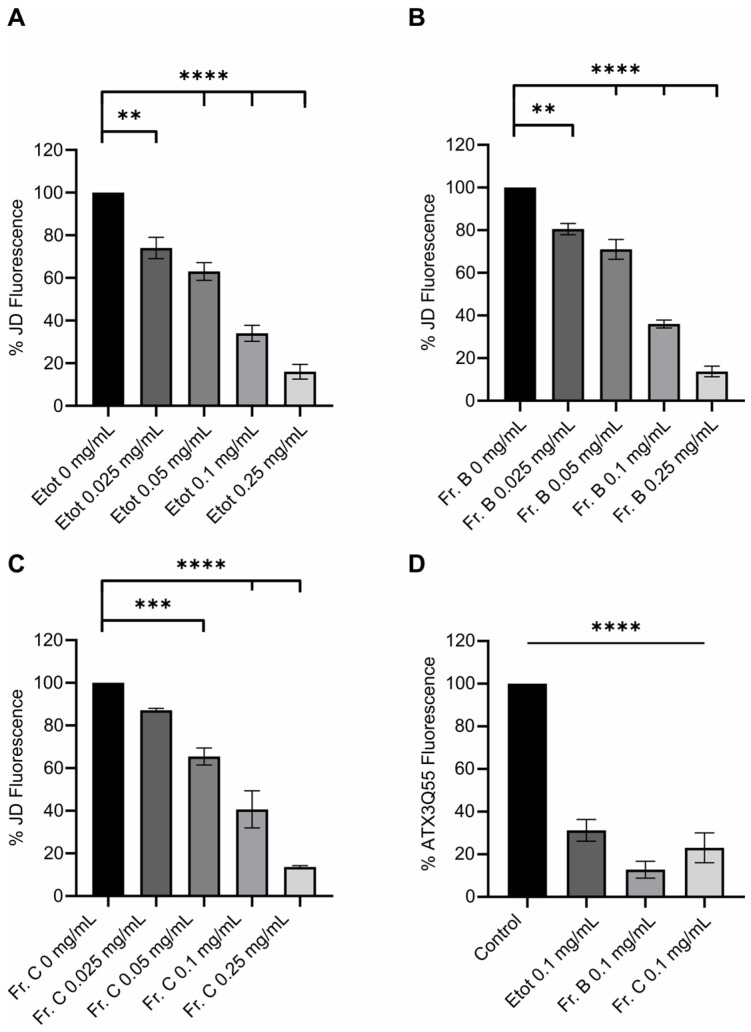
Thioflavin T assay of JD (**A**–**C**) and ATX3Q55 (**D**) incubated with different concentrations of cinnamon bud extract (Etot) and its enriched Fr. B and C. Additionally, 50 μM of purified JD protein was incubated at 37 °C in the presence of different concentrations (0, 0.025, 0.05, 0.1 and 0.25 mg/mL) of Etot (**A**), Fr. B (**B**), or C (**C**). Further, 25 μM of purified ATX3Q55 was incubated alone (control) or in the presence of 0.1 mg/mL of Etot, Fr. B, or C. ThT fluorescence was monitored for 24 h and reported as a percentage of the untreated JD (0 mg/mL) or ATX3Q55 control (**D**). Extracts incubated with ThT alone were used as controls, and their fluorescence was subtracted to correct for background. Data are represented as mean ± SEM from three technical replicates and derived from three independent experiments. Statistical analyses were assessed by a one-way factorial analysis of variance (one-way ANOVA), followed by Dunnett’s multiple comparisons test. ** *p* < 0.01; *** *p* < 0.001; and **** *p* < 0.0001.

The anti-amyloidogenic potential of cinnamon bud extract (Etot) and its two major fractions, the polyphenol-enriched fraction B (Fr. B) and the rich-in-cinnamaldehyde-and-its-derivatives fraction C (Fr. C), was initially evaluated using the Josephin domain (JD), the structured domain of the ataxin-3 (ATX3) protein. The focus on JD arises from its key role in the early steps of protein aggregation. Following native-like oligomerization, JD undergoes partial unfolding and transitions into β-sheet-rich structures, ultimately leading to the formation of oligomeric intermediates within the full-length protein [36,48]. Therefore, identifying compounds capable of interfering with this early conformational transition is particularly valuable in developing anti-aggregation strategies. To evaluate the effects of Etot and its fractions, JD aggregation kinetics were monitored using the Thioflavin T (ThT) spectrofluorimetric assay, which exploits the enhanced fluorescence of this benzothiazole dye upon binding to β-sheet-rich aggregates. Monomeric JD (50 μM) was incubated in PBS at 37 °C in the presence of ThT and either the Etot or fractions B or C at concentrations ranging from 0.025 to 0.25 mM. Fluorescence emission at 535 nm was recorded every 30 min over a 24 h period. Aware of the limitations of the ThT assay, particularly potential interference by polyphenols, we used it mainly to determine the appropriate concentrations of the extract and fractions for subsequent experiments. Figure 1A–C shows the fluorescence percentages measured after 24 h, when maximum fluorescence was reached, and normalized to the DMSO control. At this time point, the interference of the compounds incubated alone was minimal (see Figure S1). Under these conditions, Etot, Fr. B, and Fr. C showed a clear dose-dependent reduction in fluorescence, with maximum decreases of 84%, 86%, and 86% at 0.25 mg/mL, respectively. The assay was then applied to evaluate the effect of Etot and Fr. B and C on the aggregation kinetic of the expanded ATX3 carrying 55 glutamine residues (ATX3Q55) (Figure 1D). Monomeric ATX3Q55 (20 μM) was incubated in PBS at 37 °C in the presence of ThT and of Etot, Fr. B or C, at a concentration of 0.1 mg/mL and with DMSO 1% used as a control. This concentration was selected to balance the strong effect observed on JD at this concentration (about 60% reduction) with minimal interference with DMSO, which perturbs aggregation at higher concentrations. Under these conditions, a maximum reduction of 70%, 88% and 77% was observed in the presence of Etot, Fr. B or Fr. C, respectively (Figure 1D), thereby demonstrating that this dose is sufficient to induce the activity also on the full-length protein. Notably, neither fraction showed stronger inhibitory activity than the whole extract, suggesting that these components represent relevant contributors to the overall anti-amyloidogenic properties of the extract. Although cinnamaldehyde and the polyphenol-enriched fraction appear to account for a substantial part of this activity, the contribution of additional constituents present in the extract cannot be excluded. Indeed, other components within the phytochemical matrix may modulate the biological response through additive, synergistic, or antagonistic interactions. Nevertheless, considering that the tested preparation represents a nutraceutical product, its biological activity should be interpreted as an intrinsic property of the whole extract, where the combined action of multiple bioactive molecules ultimately determines the observed anti-amyloidogenic effect.

### 2.2. Solubility Assay Confirms the Capability of Cinnamon Extract and Its Fractions to Affect Ataxin-3 Aggregation

**Figure 2:**
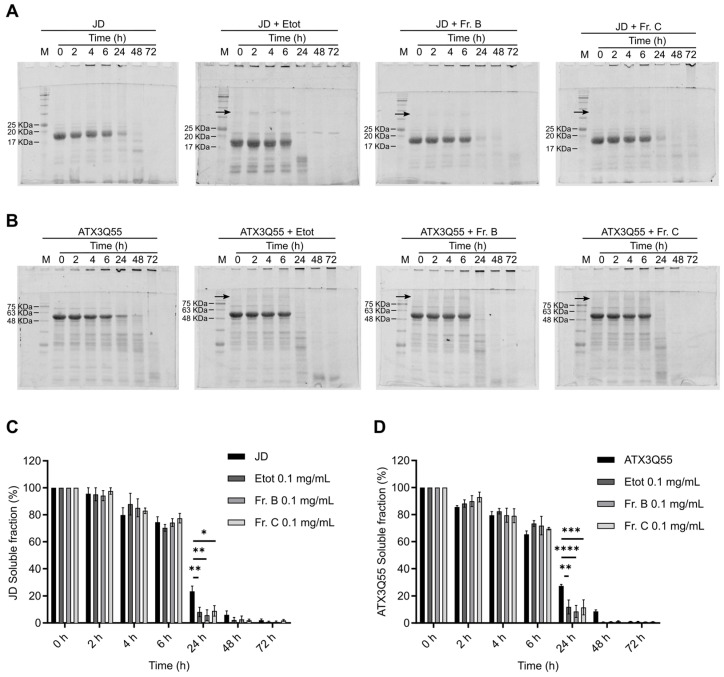
Solubility test of JD (**A**,**C**) and ATX3Q55 (**B**,**D**) incubated with different concentrations of Etot and Fr. B and Fr. C. Additionally, 50 μM and 25 μM of purified JD and ATX3Q55 proteins, respectively, were incubated at 37 °C in the presence of 0.1 mg/mL of Etot, Fr. B, or Fr. C. Protein signal quantification from SDS-PAGE gels was normalized on untreated JD (**A**) or ATX3Q55 (**B**). The arrows indicate the presence of SDS-resistant species. Data are reported as mean ± SEM from three independent experiments. Statistical analyses were assessed by a two-way factorial analysis of variance (2-way ANOVA), followed by Dunnett’s multiple comparisons test. Representative SDS-PAGE gels of JD (**C**) and ATXtQ55 (**D**) treated with 0.1 mg/mL of Etot, Fr. B, or Fr. C. * *p* < 0.05, ** *p* < 0.01; *** *p* < 0.001; and **** *p* < 0.0001.

To further characterize the effects of Etot and its fractions on ATX3 aggregation, solubility assays were performed to assess the stability and solubility of protein aggregates, key indicators of amyloidogenesis. Aliquots of monomeric JD (50 μM; Figure 2A) and full-length ATX3Q55 (25 μM; Figure 2B) were incubated with or without 0.1 mg/mL of Etot, Fr. B and Fr. C, respectively. At selected time points, samples were centrifuged at 19,000× *g* to separate soluble and aggregated fractions. SDS-PAGE analysis of the supernatants, combined with densitometry (Figure 2C,D), revealed that Etot and its fractions markedly reduced soluble protein levels after 24 h compared with the control, indicating accelerated ATX3 aggregation. The appearance of higher molecular weight bands strongly supports an off-pathway mechanism, leading to the formation of SDS-resistant soluble aggregates. Notably, these bands were reproducibly detected across all replicates with highly comparable intensity, underscoring the robustness of the observation. These findings are in strong agreement with our previous results obtained using epigallocatechin-3-gallate and cocoa extract [49,50], further supporting a shared mechanism of action. Notably, both fractions demonstrated comparable efficacy in modulating aggregation.

### 2.3. Fourier Transform Infrared Spectroscopic Analysis Reveals the Protein Structural Changes Induced by Cinnamon Extract Components on ATX3 Aggregation

**Figure 3:**
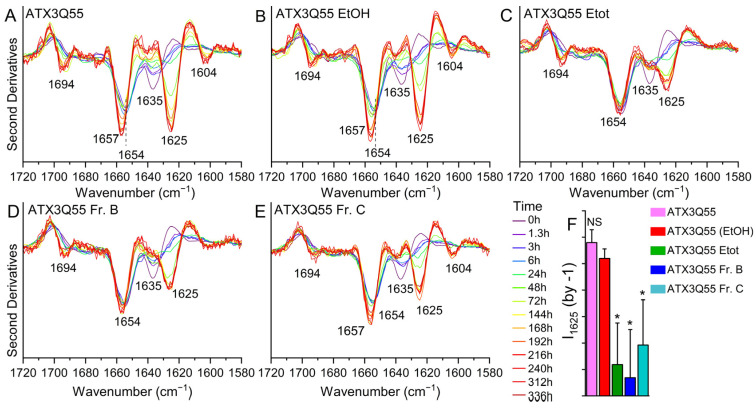
ATR-FTIR analysis of ATX3Q55 aggregation. Second-derivative spectra, calculated from absorption spectra normalized to the Amide I band area, of ATX3Q55 incubated at 37 °C for different time points: alone (**A**), in ethanol (EtOH) (**B**), in the presence of Etot (**C**), Fr. B (**D**), or Fr. C (**E**). Representative second-derivative spectra from one of three independent experiments are shown. (**F**) Intensity of the 1625 cm^−1^ band, determined from the second-derivative spectra at 336 h, shown as mean ± SD from three independent protein purifications. For clarity, second-derivative band intensities were multiplied by −1. Statistical analysis was performed by Student’s *t*-test using the ATX3Q55 (EtOH) sample as the reference group; * *p* < 0.05.

To monitor the structural changes in the ATX3Q55 samples over time, in the absence and in presence of cinnamon Etot and fractions, an Fourier transform infrared (FTIR) spectroscopic characterization was carried out. Figure 3A presents ATX3Q55 second derivative spectra within the Amide I band region, captured at various points (0 h to 14 days) during incubation at 37 °C. The Amide I band peak position is highly sensitive to the protein secondary structures, as it primarily reflects the C=O peptide bond absorption [51]. In the 0 h spectrum, the second derivative revealed two main components appearing as negative peaks: one at ~1654 cm^−1^, assignable to α-helices and random coils, and another at ~1637 cm^−1^, which, alongside a shoulder near 1690 cm^−1^, corresponds to native, intramolecular β-sheets [36,49]. In the earliest stage of incubation, a shoulder appeared around 1625 cm^−1^. This feature developed into a well-resolved peak within 24 h, quickly reaching very high intensity. Simultaneously, a distinct component emerged around 1694 cm^−1^ and intensified. Both the ~1625 cm^−1^ and ~1694 cm^−1^ components are attributed to intermolecular β-sheets, which are characteristic of protein aggregates [52,53]. Additionally, beginning at 144 h, a novel spectral component became evident near 1604 cm^−1^. This was observed alongside an upshift of the ~1654 cm^−1^ band to ~1657 cm^−1^, which concomitantly increased in intensity. These spectroscopic characteristics are assignable to C=O stretching and NH_2_ deformation modes, respectively, of glutamine side chains within amyloid-like aggregates [36,49].

Before exploring the ATX3Q55 aggregation via FTIR in the presence of the total cinnamon extract and its two fractions, we analyzed the protein aggregation in 0.7% ethanol (Figure 3B), as the Etot and the fractions for IR measurements were dissolved in this solvent. Ethanol was, indeed, employed in place of DMSO because DMSO is unsuitable for ATR measurement on hydrated protein films as it evaporates too slowly under normal conditions. As displayed, the aggregation of ATX3Q55 in ethanol closely resembled that of the protein in the absence. Figure 3C displays the second derivatives of ATX3Q55 in the presence of the Etot, from 0 to 14 days of incubation at 37 °C. What emerges is that at the end of the kinetics, the intensity of the band at ~1625 cm^−1^ is significantly lower than observed for the protein alone (see Figure 3A,B), indicating a lower amount of intermolecular β-sheet structures. Furthermore, the appearance of a resolved band at ~1604 cm^−1^ is not observed within the analyzed time-frame, indicating the absence of fibril formation.

Since the solubility assay (Figure 2) indicates an increased formation of aggregates in the presence of cinnamon extract, the reduced ~1625 cm^−1^ component, compared to ATX3Q55 alone, can be interpreted as the formation of aggregates with a more disordered internal structure and a reduced amount of intermolecular β-sheet structures in the presence of the compounds. This result resembles that found for ATX3Q55 aggregation in the presence of EGCG [49].

Quite similar results were obtained for the protein incubated with fraction B (Fr. B, see Figure 3D) and C (Fr. C, Figure 3E), though with a different intensity of the 1625 cm^−1^ peak (Figure 3F) and of the 1604 cm^−1^ component (Figure 3A–E), which was more evident for Fr. C (Figure 3E).

Figure 3F displays the mean intensity of the 1625 cm^−1^ band, primarily attributed to intermolecular β-sheets, for each protein preparation after 14 days of incubation at 37 °C. Despite the high variability, this peak shows a significantly reduced intensity in the presence of the extracts compared to the protein alone, indicating, along with the solubility data, that aggregate formation results in fewer intermolecular β-sheet structures when the compounds are present.

### 2.4. AFM Analysis Confirms That Cinnamon Bud Extract and Its Fractions Suppress Fibrillar Aggregation of ATX3Q55

**Figure 4:**
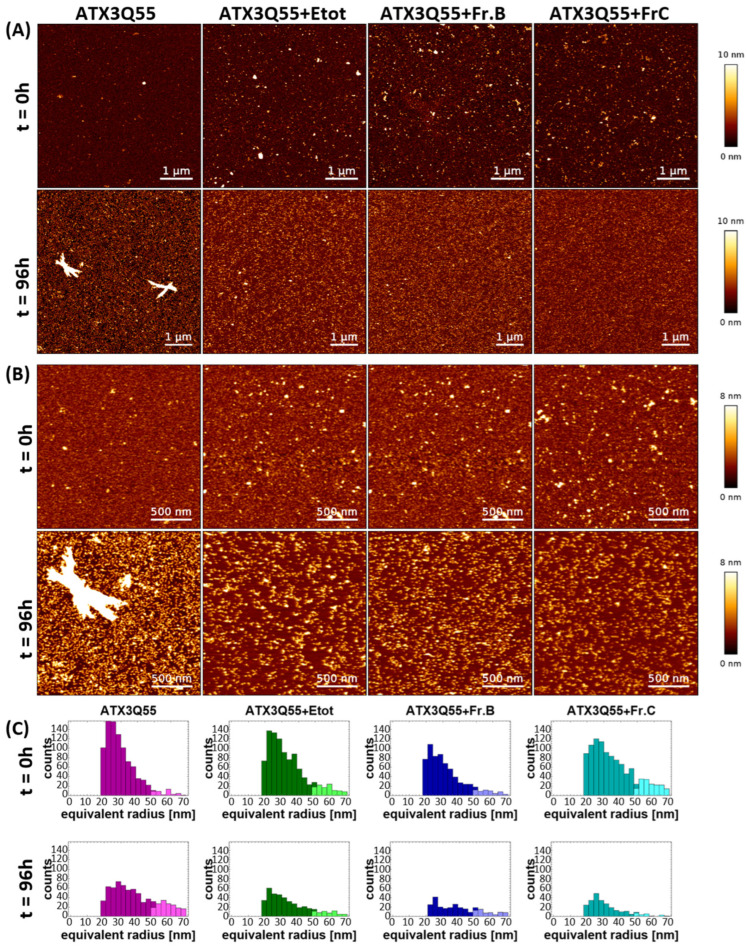
AFM analysis of ATX3Q55 aggregation in the presence of Etot, Fr. B, and Fr. C. (**A**) Representative AFM images of ATX3Q55 alone (first column), ATX3Q55 + Etot (second column), ATX3Q55 + Fr. B (third column), and ATX3Q55 + Fr. C (fourth column) at t = 0 h and t = 96 h (first and second rows, respectively). Images correspond to 6 µm × 6 µm scan areas (z-scale 10 nm). (**B**) Magnified AFM images of the same samples as in (**A**), corresponding to 3 µm × 3 µm scan areas (z-scale 8 nm). (**C**) Histograms of the equivalent-radius size distribution of detected objects larger than 20 nm at t = 0 h (upper row) and t = 96 h (lower row), obtained from the 3 µm × 3 µm AFM images. Color code: ATX3Q55 (pink), ATX3Q55 + Etot (green), ATX3Q55 + Fr. B (blue), and ATX3Q55 + Fr. C (cyan). Note that the histograms do not include the larger fibril-like objects.

Using AFM in imaging mode, a systematic study of the ATX3Q55 aggregation in the presence of Etot and Fr. B and Fr. C at different time points was conducted. Figure 4 shows representative images of samples deposited on mica immediately after sample preparation (t = 0, upper rows, Panels A and B) and after 96 h of incubation at 37 °C (lower rows). Surface height is depicted using a color-coded scale, where darker colors correspond to regions of lower height and lighter colors to regions of higher height. Panel A covers an area of 6 µm × 6 µm, whereas Panel B shows a magnified 3 µm × 3 µm view to highlight fine structural details. At t = 0, all conditions show a uniform surface with no detectable large aggregates (Figure 4A,B, upper rows). However, comparison of the samples revealed that the total extract and both fractions already promoted the formation of a greater number of small aggregates compared with the control condition (ATX3Q55 alone), suggesting an early stimulatory effect on the aggregation process. After 96 h, ATX3Q55 alone exhibits large fibril-like structures (Figure 4A,B, second row on the left in both panels), confirming the intrinsic propensity of ATX3Q55 toward fibrillar aggregation. A detailed 3D rendering of one representative fibril-like structure is shown in Supplementary Figure S2B, revealing the morphological features of these aggregates. In contrast, samples incubated with Etot, Fr. B, or Fr. C remain free of large fibrillar structures (Figure 4A,B, lower rows, second, third, and fourth columns, respectively), indicating that these compounds effectively interfere with ATX3Q55 aggregation.

To complement these qualitative observations, we performed a quantitative analysis based on image binarization (Figure S2A), allowing us to measure both the number and equivalent-radius distribution of objects on mica (see Section 3.4 for details). Figure 4C presents histograms of object size distributions for equivalent radii > 20 nm (dark colors) and >50 nm (light colors) from 3 µm × 3 µm images, comparing ATX3Q55 alone and in the presence of Etot, Fr. B, or Fr. C at t = 0 h and t = 96 h. The comparison of the data at t = 0 h (Figure 4C, first row) and t = 96 h (Figure 4C, second row) reveals the evolution in the number of smaller and larger objects over time. Histograms do not account for the larger fibril-like objects. It is important to note that the aggregation phenomena are not simply related to the total number of objects detected on the surface (or the total area of the histograms) but also to the redistribution among smaller or larger objects. Indeed, by focusing on the right-hand tails of the histograms (light colors)—corresponding to larger objects—we observe that at t = 96 h in the absence of extracts (Figure 4C, second row, pink histogram), a substantial number of objects with sizes of several tens of nanometers are present. Conversely, in the presence of Etot, Fr. B, and Fr. C (Figure 4C, second row, green, blue, and cyan histograms, respectively), the number of larger detected objects is systematically reduced, in particular, Fr. B more effectively than Etot and Fr. C.

### 2.5. STD NMR Analysis Identifies ATX3-Interacting Ligands in Cinnamon Bud Extract

In order to identify the specific components of Etot that interact with ATX3 and are thus primarily implicated in the modulation of protein aggregation propensity, Saturation Transfer Difference (STD) NMR spectroscopy was employed [54]. STD NMR represents a highly versatile and powerful approach for the characterization of transient ligand–receptor interactions, owing to its robustness, high sensitivity, and reliability. The technique is broadly applicable to the screening of individual compounds [55,56] and focused libraries of small molecules, as well as complex mixtures [50,57], particularly in the context of identifying ligands capable of interacting with amyloid species [57].

**Figure 5:**
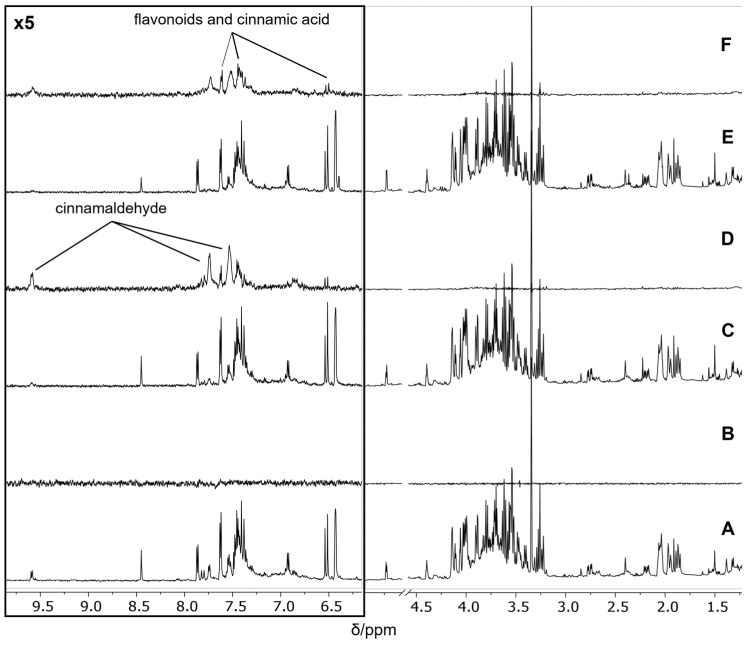
NMR binding studies with ATX3 monomers and oligomers. (**A**) ^1^H-NMR spectrum of a solution containing Etot (5 mg/mL); (**B**) Blank STD NMR spectrum of the same sample of (**A**); (**C**) ^1^H-NMR spectrum of a solution containing Etot (5 mg/mL) and ATX3 protein (10 μM) immediately after protein purification; (**D**) STD NMR spectrum of the same sample of (**C**); (**E**) ^1^H-NMR spectrum of a solution containing Etot (5 mg/mL) and ATX3 protein (8 μM) after protein incubation at 37 °C for 4 days; (**F**) STD NMR spectrum of the same sample of (**E**). Samples were dissolved in the PBS buffer, pH 7.2. STD spectra were acquired with 1024 scans and 2 s of saturation time at 600 MHz, 25 °C.

NMR spectra (Figure 5) were acquired on samples of ATX3Q55 either immediately after purification (Figure 5C,D), when the protein is predominantly monomeric, or following incubation at 37 °C for 4 days (Figure 5E,F), a condition under which ATX3 is mainly present in its oligomeric form. In both cases, the presence of ATX3 ligands was unequivocally confirmed by the observation of their characteristic resonances in the STD NMR spectra. As shown in Figure 5, the compounds directly interacting with ATX3Q55 include flavonoids, major constituents of Fr. B, and cinnamaldehyde and cinnamic acid, two structurally related molecules differing in the oxidation state of the carbonyl group and representing the most abundant components of Fr. C [28,29]. These findings may account for the significant modulation of ATX3 aggregation observed upon treatment with both Fr. B and Fr. C. Notably, the relative STD intensities of cinnamaldehyde are higher in the presence of monomeric ATX3 (Figure 5D) but significantly decrease following incubation with the oligomeric form (Figure 5F). From a structural perspective, this differential behavior indicates that the specific binding pocket or surface epitope recognized by cinnamaldehyde on the monomer surface becomes sterically hindered, buried, or conformationally rearranged during the protein self-assembly process. This suggests that cinnamaldehyde acts preferentially at the monomer level, potentially stabilizing the native state or blocking the very earliest stages of oligomerization. Conversely, cinnamic acid and flavonoids exhibit comparable STD intensities in both monomeric and oligomeric samples (Figure 5D,F), suggesting similar binding affinities across ATX3 conformational states. This structurally implies that their target binding sites remain exposed and fully accessible throughout the protein’s structural transitions. Consequently, these multi-component constituents possess the capacity to target multiple species along the aggregation pathway, interacting effectively with both early monomers and advanced oligomeric assemblies.

Taken together, these *in vitro* findings place cinnamon bud extract within the broader context of polyphenol-mediated modulation of protein aggregation. A growing body of evidence has demonstrated that isolated polyphenols, including epigallocatechin-3-gallate (EGCG), resveratrol, curcumin, quercetin, and proanthocyanidins, can interfere with amyloidogenic pathways of several disease-associated proteins by reducing nucleation and fibril growth, remodeling preformed aggregates, or redirecting misfolding pathways toward soluble and less toxic species [58,59,60]. Similarly, complex polyphenol-rich extracts derived from natural sources, including cocoa, green tea, and other plant matrices, have shown anti-aggregation activity, supporting the concept that mixtures of bioactive compounds may act through synergistic or complementary mechanisms rather than through a single active constituent [50,61,62].

Within the specific context of ATX3 aggregation, however, evidence from natural extracts remains limited. Recently, cocoa flavanol-rich extract was shown to inhibit ATX3 amyloid aggregation and ameliorate disease-associated phenotypes in a cellular and *C. elegans* model of spinocerebellar ataxia type 3 [50]. Consistently, our results demonstrate that cinnamon bud-derived fractions can modulate ATX3 aggregation by reducing β-sheet-rich assemblies, suppressing fibrillar structures, and promoting the formation of soluble non-fibrillar species. Unlike cocoa, whose activity has been mainly associated with flavanol constituents, the effects observed here likely arise from the combined contribution of multiple cinnamon-derived molecules, including procyanidins, flavonoids, cinnamic acid derivatives, and cinnamaldehyde. Thus, our findings extend the current evidence on natural polyphenol-based aggregation modulators by identifying cinnamon bud extract as an additional phytochemical source capable of interfering with ATX3 misfolding and aggregation pathways.

### 2.6. Polyphenol-Rich Cinnamon Bud Extract Ameliorates SCA3 Phenotypes in C. elegans via Locomotion Rescue and Oxidative Stress Protection

#### 2.6.1. Cinnamon Bud Polyphenol Enhances Mobility in a *C. elegans* SCA3 Model

A *Caenorhabditis elegans* model of SCA3 was employed to evaluate the potential therapeutic effects of Etot and Fr. B on disease-associated phenotypes. Fr. C was not included in these experiments due to its high cinnamaldehyde content, which has been shown to significantly alter the expression of multiple metabolic genes, particularly those involved in glutathione metabolism (gst-1, gst-2, gst-4, gst-5, gst-6, gst-7, gst-8, gst-25, gst-30, gst-38, gst-44, and gcs-1) [63]. Such transcriptional changes could independently influence oxidative stress responses and cellular homeostasis, thereby confounding the interpretation of neuroprotective effects by masking or amplifying the impact of Fr. C on SCA3-associated phenotypes. Therefore, the *in vivo* evaluation was limited to Etot and Fr. B, allowing the assessment of aggregation-related biological effects while minimizing potential confounding metabolic responses.

**Figure 6:**
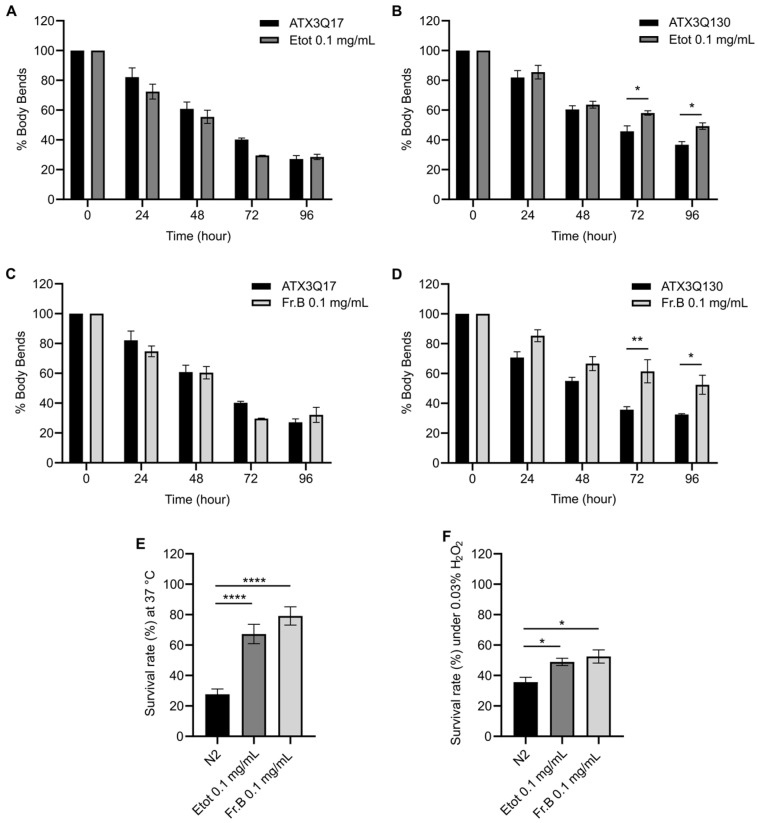
*In vivo* properties of Etot and its polyphenol-enriched Fr. B. Etot extract (**A**,**B**) and Fr. B (**C**,**D**) neuroprotective activity in *C. elegans* models of SCA3: control and ataxic strains were employed, expressing respectively the non-pathological ATX3 variant with 17Q (ATX3Q17; panels (**A**,**C**)) and the toxic form with 130Q (ATX3Q130; panels (**B**,**D**)). Synchronous 1-day adult worms were placed on a plate seeded with heat-killed OP50 in the presence or absence of 0.1 mg/mL Etot extract/Fr. B at 25 °C. Worms were transferred daily on a new plate, and body bends were counted for 20 s after 24, 48, 72, and 96 h of treatment. Data were expressed as a percentage of motility increase compared to the 0 h time point. The mean ± SEM was reported. Etot extract and Fr. B antioxidant activity in wild-type worms by heat (**E**) and oxidative (**F**) stress resistance assays. One-day adult worms were treated for 48 h with 0.1 mg/mL of both extract and fraction. The ability of treated vs. untreated animals to contrast heat stress was determined by their survival at 37 °C. Survival after the exposure to an oxidative agent (H_2_O_2_ for 2 h) allowed measurement of oxidative stress resistance of treated vs. untreated nematodes. The median lifespan of control animals at 37 °C and after 2 h exposure to H_2_O_2_ was identified, and percent survival of treated vs. untreated worms was calculated at this time point. The mean survival rate of at least three biological replicates was reported. * *p* ≤ 0.05, ** *p* ≤ 0.01 by two-way ANOVA with Sidak’s multiple comparisons test; * *p* ≤ 0.05; **** *p* ≤ 0.0001 by unpaired *t*-test.

Transgenic strains expressing in neuronal cells either the wild-type (ATX3Q17-GFP) or pathogenic (ATX3Q130-GFP) variant of human ATX3, fused to a GFP tag and expressed under the pan-neuronal *unc-119* promoter, were employed [49]. Etot and Fr. B were administered at a final concentration of 0.1 mg/mL by supplementing *Escherichia coli* OP50, the standard food source for *C. elegans*. This concentration was selected based on the *in vitro* assays, in which it showed the highest biological activity. A dose–response evaluation was also performed in wild-type N2 worms, and lifespan analysis demonstrated that treatment with either Etot or Fr. B at 0.1 mg/mL did not induce detectable toxicity under our experimental conditions (Figure S3). Higher concentrations were not evaluated because they would have required increasing the DMSO concentration; therefore, to minimize solvent exposure, the DMSO content was maintained below the 2% threshold reported to be well tolerated by *C. elegans* [64]. Phenotypic analyses were performed up to 96 h post-treatment, including lifespan assessment and evaluation of motor function, measured by body bend frequency (Figure 6). None of the tested compounds produced a statistically significant extension of lifespan (Figure S3). However, after 72 h of treatment, both Etot and Fr. B significantly improved locomotor function in the ATX3Q130-GFP strain compared to untreated controls, with body bend frequencies increasing by 12% and 26%, respectively (Figure 6B,D). These improvements were maintained at 96 h, showing similar enhancements (Figure 6B,D). No significant changes were observed in the ATX3Q17-GFP strain (Figure 6A,C), confirming that the effect is specific to the ataxic phenotype. rather than a general consequence of heterologous protein expression.

#### 2.6.2. Oxidative Stress and the Potential Protective Role of Cinnamon Bud Extract in SCA3

Oxidative stress is a key contributor to SCA3 pathogenesis. Expanded ATX3 impairs mitochondrial function, increasing ROS production and promoting lipid peroxidation, DNA oxidation, and protein misfolding [42,44,45]. Furthermore, oxidative stress further compromised the ubiquitin-proteasome system and autophagy, hindering the clearance of mutant ATX3 and amplifying protein aggregation and cellular toxicity [46,47]. Conversely, the accumulation of misfolded and aggregated ATX3 further exacerbates oxidative stress by disrupting mitochondrial homeostasis and proteostasis, establishing a self-perpetuating pathogenic cycle in which oxidative stress promotes ATX3 aggregation, while protein aggregation further enhances oxidative damage [42]. Interrupting this positive feedback loop therefore represents an attractive therapeutic strategy. To elucidate whether the beneficial effect of Etot and its polyphenol-enriched Fr. B is due not only to the direct alteration of ATX3 aggregation but also to a reduction in oxidative stress, stress-resistance assays were performed in the *wild-type* N2 *C. elegans* strain. One-day-old adult worms were pre-treated for 48 h with 0.1 mg/mL of Etot or Fr. B and then exposed to thermal and oxidative stress. A heat stress assay was used as an indirect indicator of neuronal dysfunction and impaired proteostasis in *C. elegans* [65]. For thermal stress, animals were exposed to 37 °C, and survival was assessed. Median lifespan under control conditions was reached within 5 h of heat exposure. This point was used to compare survival rates across treatments (Figure 6E). Both Etot and Fr. B significantly increased thermal stress resistance, improving survival by 40% and 52%, respectively. For oxidative stress, worms were exposed to 0.03% H_2_O_2_ on an NGM plate seeded with live *E. coli* OP50. Survival was monitored hourly over a 2 h period until all animals had died. Etot and Fr. B significantly enhanced oxidative stress resistance, increasing survival by 13% and 17%, respectively (Figure 6F), consistent with previous reports on bioactive compounds in *C. elegans* [66].

Overall, our findings support the hypothesis that the beneficial effects of the cinnamum bud extract result from a dual mechanism of action, combining the direct modulation of ATX3 aggregation with the attenuation of oxidative stress. By acting on both processes, the extract may interrupt the pathogenic positive feedback loop linking protein aggregation and oxidative stress, thereby reducing the cellular damage associated with mutant ATX3.

## 3. Materials and Methods

### 3.1. Cinnamon Bud Extract and Polyphenol-Enriched Fractions

Cinnamon bud extract was obtained by ultrasound-assisted hydroalcoholic extraction, whereas polyphenol-enriched fractions were obtained by reverse-phase C18 flash chromatography as described in Ciaramelli et al. (2022) [28]. The same study also characterized the metabolic profile of the buds using nuclear magnetic resonance (NMR) spectroscopy and ultra-performance liquid chromatography coupled with high-resolution mass spectrometry (UPLC-HR-MS), highlighting the enrichment in B-type procyanidins and glycosylated flavonoids [28].

### 3.2. Protein Expression and Purification

The JD and ATX3Q55 proteins were expressed through the pET21-a inducible system using the *Escherichia coli* BL21 Tuner (DE) as the expression strain [67]. The purification procedures are reported in Amigoni et al. (2019) [67] and Bonanomi et al. (2014) [49] for JD and ATX3Q55, respectively. Cells were grown at 37 °C in LB low-salt–ampicillin medium and induced with 0.5 mM IPTG at OD600 0.8 for 3 h for JD and 45 min for ATX3Q55 at 30 °C. Protein purification was performed using HisPur™ Cobalt Resin (Thermo Fisher Scientific, Rockford, IL, USA) for the affinity chromatography and loading purified fractions onto a Superose 12 10/300 GL gel filtration column (GE Healthcare, Life Sciences, Little Chalfont, UK) to isolate the JD and ATX3Q55 monomeric form.

### 3.3. Thioflavin T Assay

The JD and ATX3Q55 aggregation kinetics in the presence of the extracts were followed by monitoring the Thioflavin T (ThT) fluorescence as previously described [50]. Briefly, different concentrations of both total extract (Etot) and its fractions (Fr. B and Fr. C) were incubated at 37 °C for 24 h in PBS pH 7.2 with 50 μM of freshly purified JD protein and in the presence of ThT 20 μM (Sigma-Aldrich, St. Louis, MO, USA). The ATX3Q55 protein was used at 25 μM and tested with 0.1 mg/mL of both the Etot and its enriched fractions. The use of a lower concentration of AT3Q55 reflects the higher aggregation propensity of the full-length protein compared to the JD alone. The control samples were incubated with DMSO in the same amount as the treated samples. Data were expressed as a percentage relative to the untreated samples and were reported as mean ± SEM of the maximum ThT fluorescence values reached. The experiments were replicated at least three times.

### 3.4. Solubility Test

A solubility test was performed as described before [50]. Briefly, ATX3Q55 and JD proteins were incubated at 25 and 50 μM, respectively, in PBS pH 7.2 in the presence of 0.1 mg/mL of both Etot and its fractions B and C. For the untreated samples, DMSO was added to reach the same amount of DMSO as the treated samples. Aliquots at different incubation times (0 h, 2, 4, 6, 24, 48 and 72 h) were centrifuged at 19,000× *g* for 15 min, and 3 μL of the supernatants were loaded on SDS-PAGE performed with 10% (ATX3Q55) or 14% (JD) polyacrylamide gel. EZBlue Gel Staining Reagent (Sigma-Aldrich, St. Louis, MO, USA) was used to stain the gels, and densitometric analysis was performed using the Image Studio software 5.2 (LI-COR Biosciences, Lincoln, NE, USA). Data was reported as a percentage and normalized to the time 0 h of the control. At least three independent experiments were performed.

### 3.5. Fourier Transform Infrared (FTIR) Spectroscopy

For FTIR measurements in attenuated total reflection (ATR), 2 μL of each protein sample was placed on a diamond plate within a single-reflection ATR device (Quest, Specac, Mount Kisco, NY, USA). After the solvent evaporated, spectra were recorded and analyzed as previously detailed [52,53]. A Varian 670-IR spectrometer (Varian Australia Pty Ltd., Mulgrave, VIC, Australia) was used with the following settings: 1024 scan co-additions, 2 cm^−1^ spectral resolution, 25 kHz scan speed, triangular apodization, and a nitrogen-cooled Mercury Cadmium Telluride (MCT) detector. The spectra were collected from three independent experiments, each performed on a different protein purification.

Before analysis, buffer absorption (collected under identical conditions to the protein samples) and, if needed, residual vapor peaks were subtracted from the absorption spectra. The spectra were then smoothed using the Savitsky–Golay method (25 points). Finally, after normalization of the spectra at the Amide I band area, second-derivative calculations were performed using Resolutions-Pro software 5.2.0 (Varian Australia Pty Ltd.). Second derivatives are useful for observing spectral changes, provided that the same analytical methods are applied to the original data and that the initial absorption spectra are of high quality [68].

### 3.6. Atomic Force Microscopy (AFM)

The AFM imaging procedure adopted to characterize the ATX3Q55 stage of aggregation has been previously described [69,70]. Briefly, 5 µL of each 25 μM ATX3Q55 sample was collected at two incubation times (0, 96 h), diluted 1:4 in PBS, and incubated for 5 min on a freshly cleaved mica substrate with a final volume of 20 µL. Similarly, ATX3Q55 was incubated at 25 μM with Etot, Fr. B, and Fr. C at a final concentration of 0.1 mg/mL at 37 °C and analyzed at the same timepoints (0, 96 h). After incubation, samples were rinsed 3 times with 1 mL of MilliQ water and dried under a gentle nitrogen flow.

All the Atomic Force Microscopy (AFM) measurements were performed in Tapping Mode in air using stiff silicon cantilevers (RTESP-Bruker, Billerica, MA, USA, resonant frequencies ∼300 kHz, spring constant ∼40 N/m). AFM images (6 μm × 6 μm) were acquired with a JPK Nanowizard 4XP (Bruker Nano GmbH, Berlin, Germany) at a scan rate of 1 Hz with 1024 × 1024 pixels resolution. The AFM images were analyzed using the commercial JPK Image Processing software 8.1.70 and then by a customized image analysis software [71] in Mathematica™ 12.1 capable of detecting the number and the equivalent radius of objects in each image. A crucial step of the analysis is the binarization of the images. A representative example of the binarization is shown in Figure S2A. All data in this study were verified by sampling a wide range of areas over the sample surfaces.

### 3.7. NMR-Binding Studies

Cinnamon total extract (Etot), dissolved in deuterated PBS (d-PBS) 10 mM (5 mg/mL) was added to an aliquot of ATX3Q55, reaching the final concentration of 5 mg/mL and 10 μM, respectively. The pH of samples was measured with a Microelectrode (InLab Micro, Mettler Toledo, Columbus, OH, USA) and adjusted to pH 7.4 with NaOD and/or DCl. All pH values were corrected for the isotope effect. Experiments were run on an AVANCE III 600 MHz NMR spectrometer (Bruker, Billerica, MA, USA) equipped with a TXI (^1^H, ^13^C, ^15^N, and ^2^H lock) probe. A basic sequence from the Bruker library was used for the STD experiments. A train of Gaussian-shaped pulses of 50 ms each was employed to saturate the protein envelope selectively; the total saturation time of the protein envelope was adjusted to the number of shaped pulses and set at 2 or 3 s. On- and off-resonance spectra were acquired in an interleaved mode with the same number of scans. The STD NMR spectrum was obtained by subtracting the on-resonance spectrum from the off-resonance spectrum.

### 3.8. C. elegans Strains Maintenance and Synchronization

The following strains were used in this work: wild-type N2 Bristol, ATX3Q17, and ATX3Q130. N2 was provided by the Caenorhabditis Genetics Center (CGC, Minneapolis, MN, USA), University of Minnesota, while ATX3Q17 and ATX3Q130 were previously derived by microinjection in a *lin-15(n765ts) C. elegans* strain [49].

All the strains were maintained on Nematode Growth Medium (NGM) plates (2.5 g/L bacteriological peptone, 3 g/L NaCl, 17 g/L agar, 1 mM CaCl_2_, 1 mM MgSO_4_, 1 mM cholesterol, 25 mM KH_2_PO_4_) seeded and spread with alive *Escherichia coli* OP50 at the OD_600_ = 0.3. N2 were kept at 20 °C while ATX3Q17 and ATX3Q130 were cultured at 25 °C. All the experiments were performed starting from a synchronous population of worms on the first day of adulthood (day 0): briefly, gravid adult worms were allowed to lay eggs for 16 h and then sacrificed. Newly laid eggs grew and developed, giving rise to a synchronous population of 1-day adults after 3 days. The same procedure was applied at both 20 °C and 25 °C.

### 3.9. Body Bends Assay

20 fluorescent 1-day adult ATX3Q17 and ATX3Q130 synchronized worms were selected and placed on fresh plates seeded with heat-killed OP50 in the presence or absence of 0.1 mg/mL Etot extract or its polyphenol-enriched Fr. B. Dead OP50 was obtained by heating up alive OP50 OD_600_ = 0.3 at 65 °C for 1 h. Body bends per minute were counted under a microscope (Leica MZ FLIII, Leica Microsystem, Wetzlar, Germany) every 24 h for 5 days of treatment after moving nematodes onto a new plate. The percentage of motility in the presence of the extract and its fraction was calculated for each time point. Data from three biological replicates were represented.

### 3.10. Thermal Stress Resistance Assay

Synchronized 1-day adult N2 were pre-treated for 48 h on NGM plates seeded with heat-killed *E. coli* OP50 in the presence or absence of 0.1 mg/mL Etot extract or Fr. B. Additionally, 30 worms per each group were incubated at 37 °C for 5 h on NGM plates seeded with alive *E. coli* OP50 OD_600_ = 0.3 and pre-heated at 37 °C for 20 min. Survivals were scored every hour using a stereo microscope (Leica MZ FLIII, Leica Microsystem), and the mean ± SEM of the survival percentage of at least three biological replicates was represented.

### 3.11. Oxidative Stress Resistance Assay

Synchronous 1-day adult N2 were placed on NGM plates containing 0.1 mg/mL Etot extract or Fr. B and seeded with heat-killed *E. coli* OP50 for 48 h. At the end of the 48 h pre-treatment, 30 worms were transferred to NGM plates added with a 0.03% final concentration of H_2_O_2_ (Sigma-Aldrich/Merck, St. Louis, MO, USA) and seeded with alive *E. coli* OP50 OD_600_ = 0.3. Nematode survival after 2 h of H_2_O_2_ exposure was scored every hour under a stereo microscope. The mean ± SEM of the survival percentage of at least three biological replicates was reported.

### 3.12. Statistical Analysis

GraphPad Prism 8.0.2 software was used for statistical analyses. The statistical analysis of Tht and solubility tests were performed using the one-way ANOVA and two-way ANOVA, respectively, both followed by Dunnett’s multiple comparisons test. Statistical significance of the body bends assay was determined using two-way ANOVA and Sidak’s multiple comparisons test; an unpaired *t*-test was applied in thermal and oxidative stress resistance assays.

## 4. Conclusions

Numerous studies have shown that polyphenolic compounds, beyond their well-known antioxidant properties, exert anti-amyloidogenic activity against several proteins implicated in neurodegenerative diseases [72,73,74]. Among these compounds, procyanidins represent particularly interesting candidates due to their structural complexity, biological activity and previously reported neuroprotective effects [28,29,30]. This prompted us to investigate cinnamon buds as a natural source of bioactive molecules capable of interfering with ATX3 pathogenic aggregation, one of the key pathological hallmarks of spinocerebellar ataxia type 3. Through a combination of biochemical and biophysical approaches, it was demonstrated that both the polyphenol-enriched fraction (Fr. B) and the cinnamaldehyde-rich fraction (Fr. C) contribute to the anti-amyloidogenic properties of the total extract by inhibiting the formation of β-sheet-rich aggregates of both the Josephin domain and the full-length expanded ATX3Q55, while promoting the accumulation of soluble, SDS-resistant, and non-fibrillar assemblies. The convergence of kinetic, biochemical, structural, and morphological evidence presented here indicates that these aggregates are distinct from canonical amyloid intermediates and mature fibrils, supporting the conclusion that cinnamon-derived compounds redirect ATX3 aggregation toward an off-pathway route rather than simply delaying fibril formation. STD NMR profiling identified flavonoids, cinnamaldehyde, and cinnamic acid as direct interactors of ATX3 in the different aggregation states. Consistently, AFM confirmed the ability of the Etot and both fractions to suppress the formation of large fibrillar assemblies, supporting their role in remodeling the ATX3 aggregation pathway.

Although the biophysical experiments were performed under simplified cell-free conditions, the relevance of this aggregation-modulating activity was further supported by complementary experiments in the *C. elegans* SCA3 model, demonstrating that the molecular effects observed *in vitro* translate into beneficial phenotypic outcomes in a living organism. In this model, both Etot and Fr. B (Fr. C was not evaluated because cinnamaldehyde strongly altered the expression of glutathione-related metabolic genes [63]) significantly improved locomotor deficits associated with expanded ATX3 expression, without affecting the motility of worms expressing the non-pathogenic ATX3Q17. Moreover, these treatments enhanced resistance to thermal and oxidative stress, suggesting that cinnamon-derived compounds provide broader cytoprotective effects beyond the direct modulation of protein aggregation by influencing cellular stress responses and redox homeostasis.

Taken together, our findings indicate that cinnamon bud extract, particularly its polyphenol-enriched fraction, exerts a dual protective action by directly remodeling ATX3 aggregation toward structurally distinct off-pathway assemblies while simultaneously enhancing cellular stress resistance. These results identify cinnamon bud as promising sources of bioactive compounds with potential neuroprotective properties in the context of SCA3 and other polyglutamine disorders. Future investigations in more advanced preclinical models will be necessary to confirm these effects, evaluate their translational relevance, and determine whether these natural compounds may contribute to the development of nutraceutical-based approaches for human neurodegenerative disorders.

## Data Availability

All data supporting the findings of this study are included within the article and its Supplementary Materials.

## Supplementary Materials

The following supporting information can be downloaded at: https://www.mdpi.com/article/10.3390/molecules31142510/s1, Figure S1. Representative ThT fluorescence profiles of Cinnamomum cassia bud extracts on JD and ATX3Q55 aggregation. Figure S2. Supplementary AFM characterization and surface analysis. Figure S3. Representative survival curves of nematode strains N2 (A), ATX3Q17 (B) and ATX3Q130 (C). Table S1: Evaluation of cinnamon bud extract and fraction B on nematode lifespan.
