# Supplementary material for: Polyphenol-Rich Cinnamon Bud Extract Affects Ataxin-3 Aggregation and Ameliorates SCA3 Phenotypes Through a Dual Anti-Amyloidogenic and Antioxidant Mechanism
Source: Molecules. 2026 Jul 17;31(14):2510. doi: 10.3390/molecules31142510 (PMC13415171; doi:10.3390/molecules31142510)
Supplement: Supplementary file 1 [file molecules-31-02510-s001.zip › Figure S3 and Table S1.pdf]

Supplementary Fig. S3 and Table S1: Evaluation of cinnamon bud extract and fraction B on nematode lifespan

A

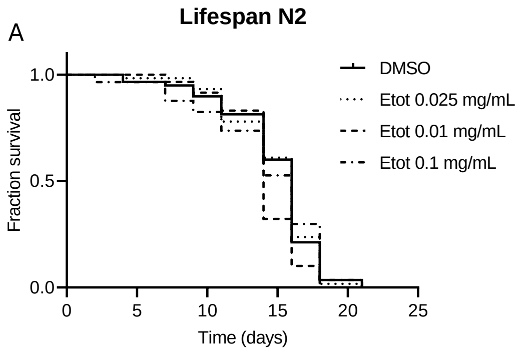

	Treatment condition	N° of worms *per* experiment	Median Life Span	Max Life Span	P-value	N° of experime nt
	DMSO	30	6.5±0.87	10.7±1.53		3
ATX3Q17	Etot	30	5.7±1.52	9.7±1.53	ns	3
	Fr. B	30	5.8±1.75	11.3±1.15		3
	DMSO	30	3.8±0.17	9.00±0.41		3
ATX3Q130	Etot	30	4.8±0.61	8.67±0.33	n.s	3
	Fr. B	30	3.5±0.33	8.00±0.58		3
	DMSO	60	14.3±0.81	19.2±1.83		3
	Etot 0,01 mg/mL	60	14.0±0.00	19.33±2.31		3
N2	Etot 0,025 mg/mL	60	15±1.41	18±0.00	n.s.	3
	Etot 0,1 mg/mL	60	14.0±0.00	20.7±0.58		3

B

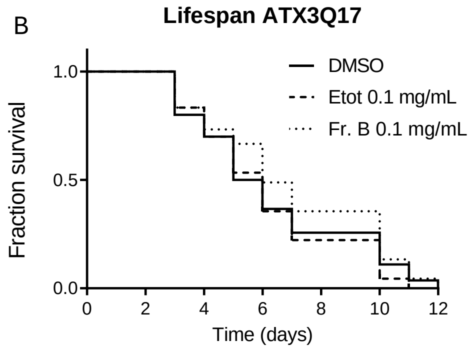

C

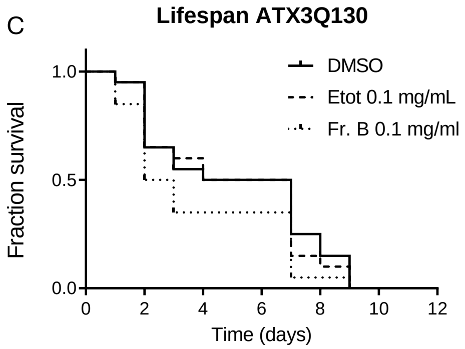

Representative survival curves of nematode strains N2 (A), ATX3Q17 (B) and ATX3Q130 (C).

Sixty (for N2) and thirty (for ATX3Q17 and ATX3Q130) synchronized nematodes at day-1 of adulthood were moved on NGM agar plates seeded with heat-killed OP50 at OD600=0,3, in the presence of the cinnamon buds components. Every two days living nematodes were transferred in a new plate with the same treatment, until the entire population is dead. Statistical analysis performed using the Logrank test (GraphPad Prism software, version 8.0.2) shows no differences in both median and maximum life span values in all the strains tested (Table 1), both with the total extract and the fraction B.
