# Supplementary figures and images for: Polyphenol-Rich Cinnamon Bud Extract Affects Ataxin-3 Aggregation and Ameliorates SCA3 Phenotypes Through a Dual Anti-Amyloidogenic and Antioxidant Mechanism
Source: Molecules. 2026 Jul 17;31(14):2510. doi: 10.3390/molecules31142510 (PMC13415171; doi:10.3390/molecules31142510)

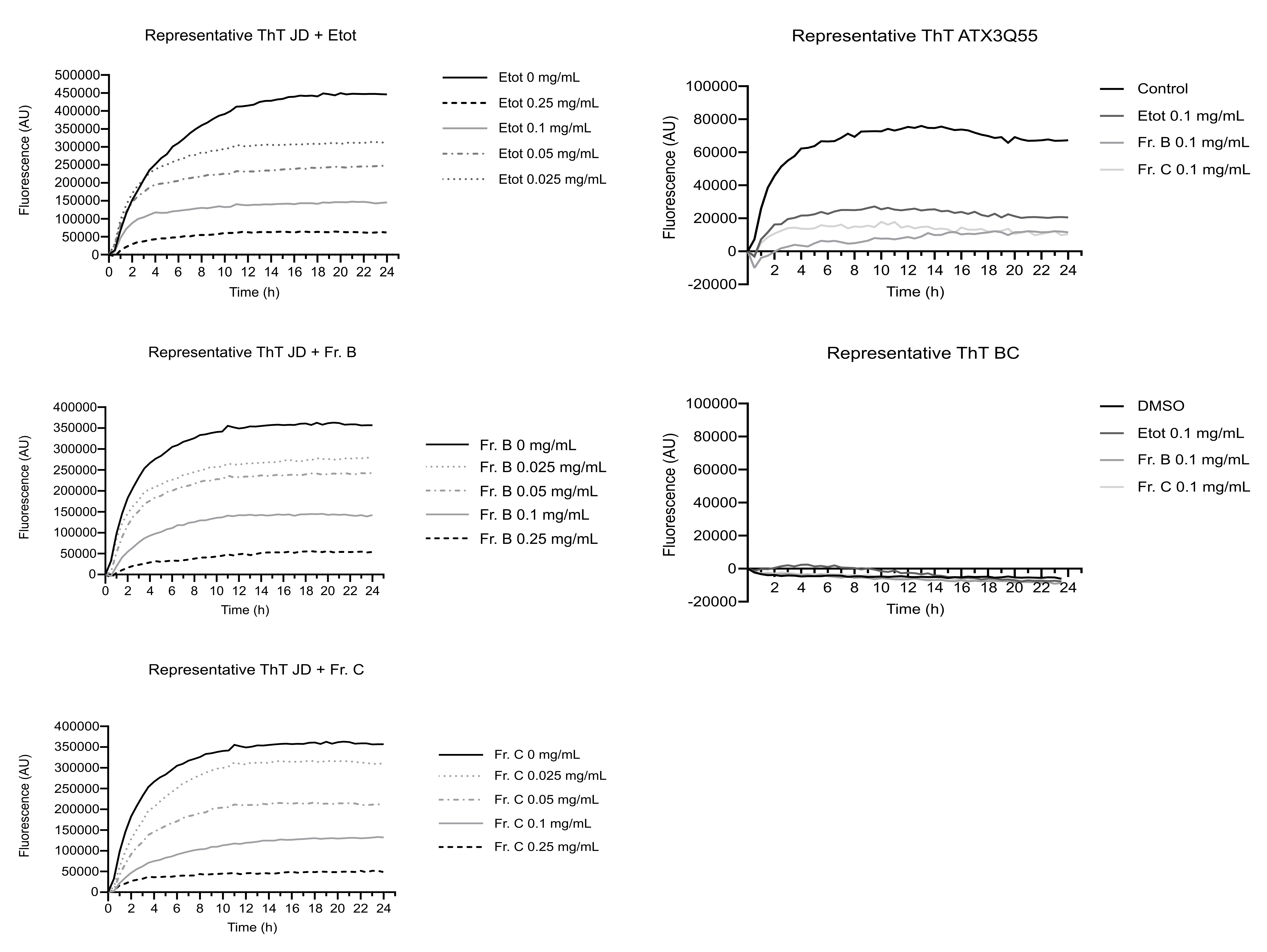

Supplement: Supplementary file 1 [file molecules-31-02510-s001.zip › Figure S1.png]

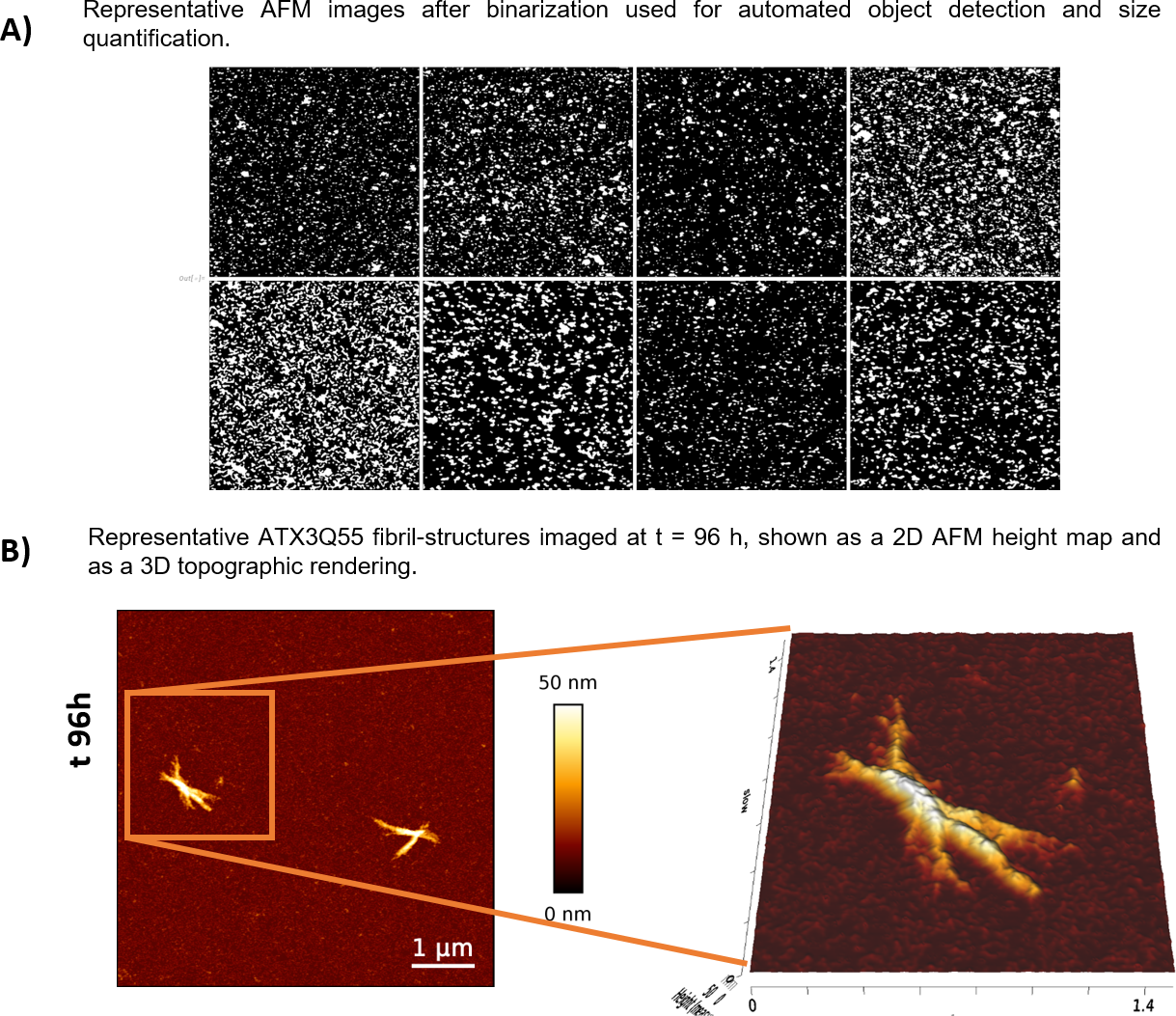

Supplement: Supplementary file 1 [file molecules-31-02510-s001.zip › Figure S2.png]
